# Amoxicillin-Clavulanic Acid-Induced Rash in Epstein-Barr Virus Infection: A Case Report of a Diagnostic Pitfall in a 24-Year-Old Male

**DOI:** 10.7759/cureus.89023

**Published:** 2025-07-29

**Authors:** Tamari Chaprava, Khatia Ostatishvili, Nana Kirtadze, Alexandre Goginava, Irakli Tabatadze

**Affiliations:** 1 Infectious Diseases, Pineo Medical Ecosystem, Tbilisi, GEO; 2 Department of Microbiology, Tbilisi State Medical University, Tbilisi, GEO; 3 Infectious Diseases, German Hospital, Tbilisi, GEO; 4 Infectious Diseases, Globalmed University Clinic, Tbilisi, GEO

**Keywords:** amoxicillin-clavulanic acid, epstein-barr virus (ebv), glucocorticosteroids, hepatosplenomegaly, infectious mononucleosis (im), maculopapular rash, tonsillitis

## Abstract

Infectious mononucleosis (IM), caused by the Epstein-Barr virus (EBV), is primarily a self-limiting illness. However, it often mimics bacterial infections, such as bacterial tonsillitis, which can represent a diagnostic pitfall and, consequently, increase the risk of developing complications. The aim of this case report is to highlight the complications resulting from the incorrect differential diagnosis of EBV-induced IM and its clinical significance. We present the case of a 24-year-old male who was initially misdiagnosed with bacterial tonsillitis. Amoxicillin-clavulanic acid was initiated for treatment, after which he developed a widespread maculopapular rash. Acute EBV infection was diagnosed via serological tests. His condition improved following the discontinuation of antibiotic therapy and the administration of supportive symptomatic treatment. This case underscores the critical need for careful differential diagnosis between viral and bacterial tonsillitis. It demonstrates that an amoxicillin-clavulanic acid-induced rash can serve as an important clue for underlying EBV infection, thereby preventing unnecessary antibiotic use and enabling the correct direction of investigation and management.

## Introduction

Infectious mononucleosis (IM) is a viral infection mainly caused by the Epstein-Barr virus (EBV). It is a contagious illness that primarily affects adolescents and young adults, although it can occur at any age. It is characterized by symptoms such as fever, sore throat, swollen lymph nodes, and fatigue, occasionally lasting for several weeks [1]. Pharyngitis (usually subacute in onset), fever, and lymphadenopathy constitute the classic triad of presenting signs [2]. EBV is primarily transmitted through oral secretions, with deep kissing identified as the major source in adolescents. Transmission in younger children can also occur via intimate contact or sharing food and utensils. Unlike many acute viral illnesses, primary EBV infection has an unusually long incubation period of approximately six weeks. Diagnosis is typically confirmed by heterophile antibody tests or EBV-specific antibody tests. Long-term consequences of EBV acquisition may include nasopharyngeal carcinoma and lymphomas [3]. However, the clinical manifestations of IM can vary widely, ranging from mild presentations to severe cases accompanied by various complications affecting multiple organ systems. These atypical manifestations include, but are not limited to, airway obstruction, pneumonia, acute myocarditis, hematological abnormalities (e.g., thrombocytopenia, hemolytic anemia, agranulocytosis), neurological disorders, hepatic involvement, and other complex inflammatory or autoimmune conditions [4].

Despite these varied presentations, IM is frequently misdiagnosed as bacterial tonsillitis due to overlapping clinical symptoms such as pharyngitis, fever, and lymphadenopathy [5]. Acute tonsillitis, a condition frequently mistaken for IM, is predominantly caused by various viruses, including double-stranded DNA viruses (such as human adenoviruses and Epstein-Barr virus) and single-stranded RNA viruses (like influenza, rhinoviruses, and enteroviruses). Conversely, the primary bacterial pathogen responsible for tonsillitis is Group A beta-hemolytic *Streptococcus* (GABHS) or *Streptococcus pyogenes* [6]. This diagnostic challenge often leads to inappropriate antibiotic prescriptions, potentially complicating the patient's clinical course. A notable complication in this context is the development of a characteristic maculopapular rash following aminopenicillin administration (e.g., amoxicillin or ampicillin), a frequent occurrence in patients with underlying EBV infection [7]. This amoxicillin-induced skin rash in IM is a well-recognized and relatively common beta-lactam-induced adverse drug reaction, typically presenting as maculopapular exanthems. While the exact mechanism is unclear and debated (true allergy, virus-dependent, or transient drug intolerance), the rash frequently appears a few days after antibiotic initiation. Although skin eruptions in acute IM can occur without antibiotic intake (incidence 4.2-13%), the incidence significantly rises to 27.8-69% following amoxicillin intake [8]. 

This case report details the presentation and management of a 24-year-old male with EBV infection, initially misdiagnosed as bacterial tonsillitis, highlighting the subsequent development of an amoxicillin-clavulanic acid-induced rash. We present a case that highlights this diagnostic pitfall, in which amoxicillin-clavulanic acid administration in a misdiagnosed case of IM led to a characteristic rash and severe airway compromise.

## Case presentation

A 24-year-old male, previously healthy and with no known history of chronic medical conditions, presented to the clinic with febrile temperatures, odynophagia, and dysphagia. Symptom onset occurred 10 days prior to hospitalization, with progressive worsening despite receiving amoxicillin-clavulanic acid therapy (1000 mg, twice daily). Initially, given the common overlap in clinical symptoms between IM and bacterial tonsillitis, an initial diagnosis of bacterial tonsillitis was made, leading to the prescribed antibiotic therapy. Documentation of investigations supporting this initial diagnosis was not available. On the fifth day of antibiotic treatment, a maculopapular rash developed, initially noted on the trunk and subsequently spreading to the face and upper and lower extremities. Given the persistence of high fever, the emergence of the rash, and the patient's deteriorating overall condition, admission to the hospital for comprehensive assessment and management was deemed necessary.

**Figure 1:**
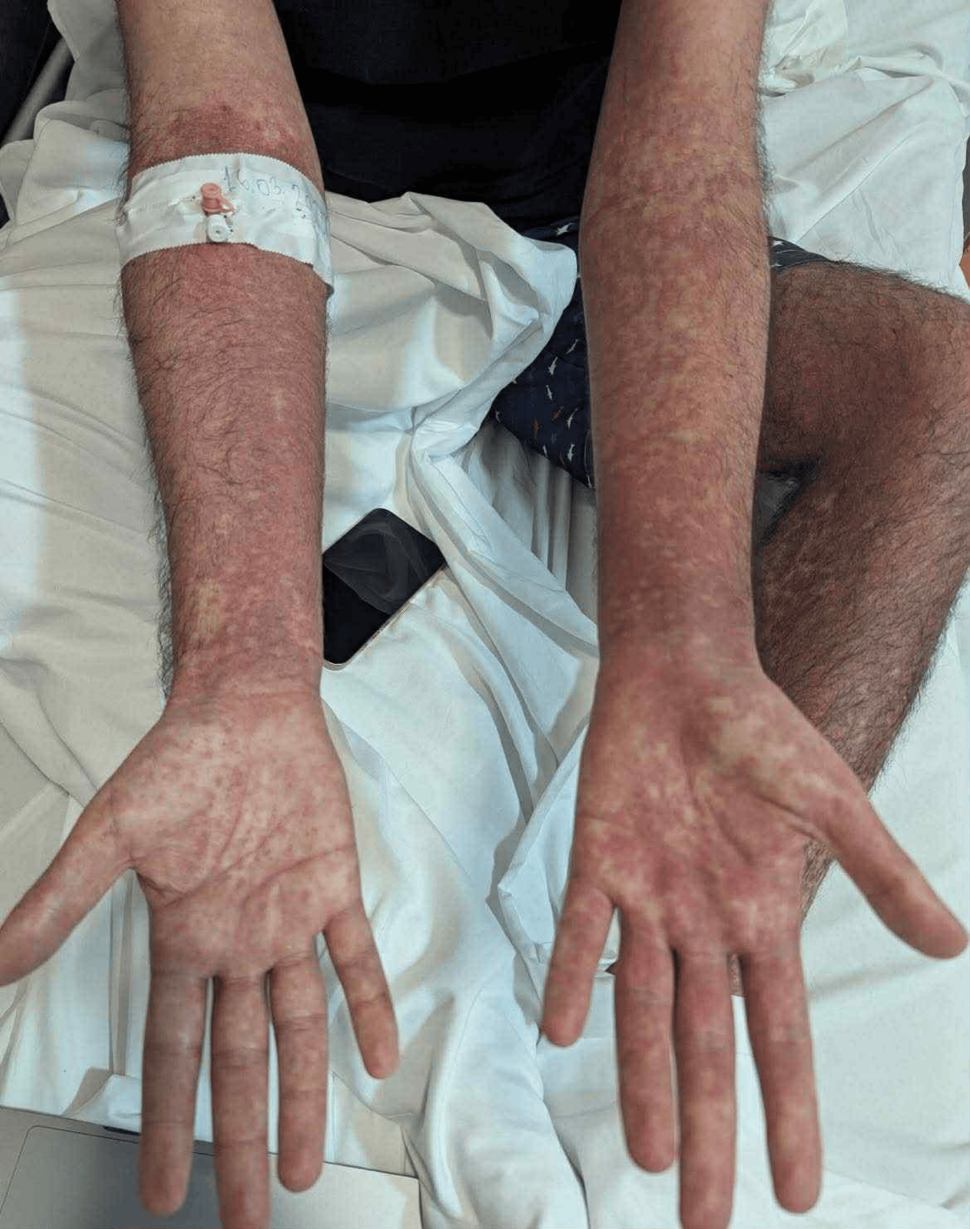
Rash that developed on the upper limbs Erythematous maculopapular rash observed on the upper limbs in a 24-year-old male patient diagnosed with infectious mononucleosis caused by the Epstein-Barr virus (EBV)

**Figure 2:**
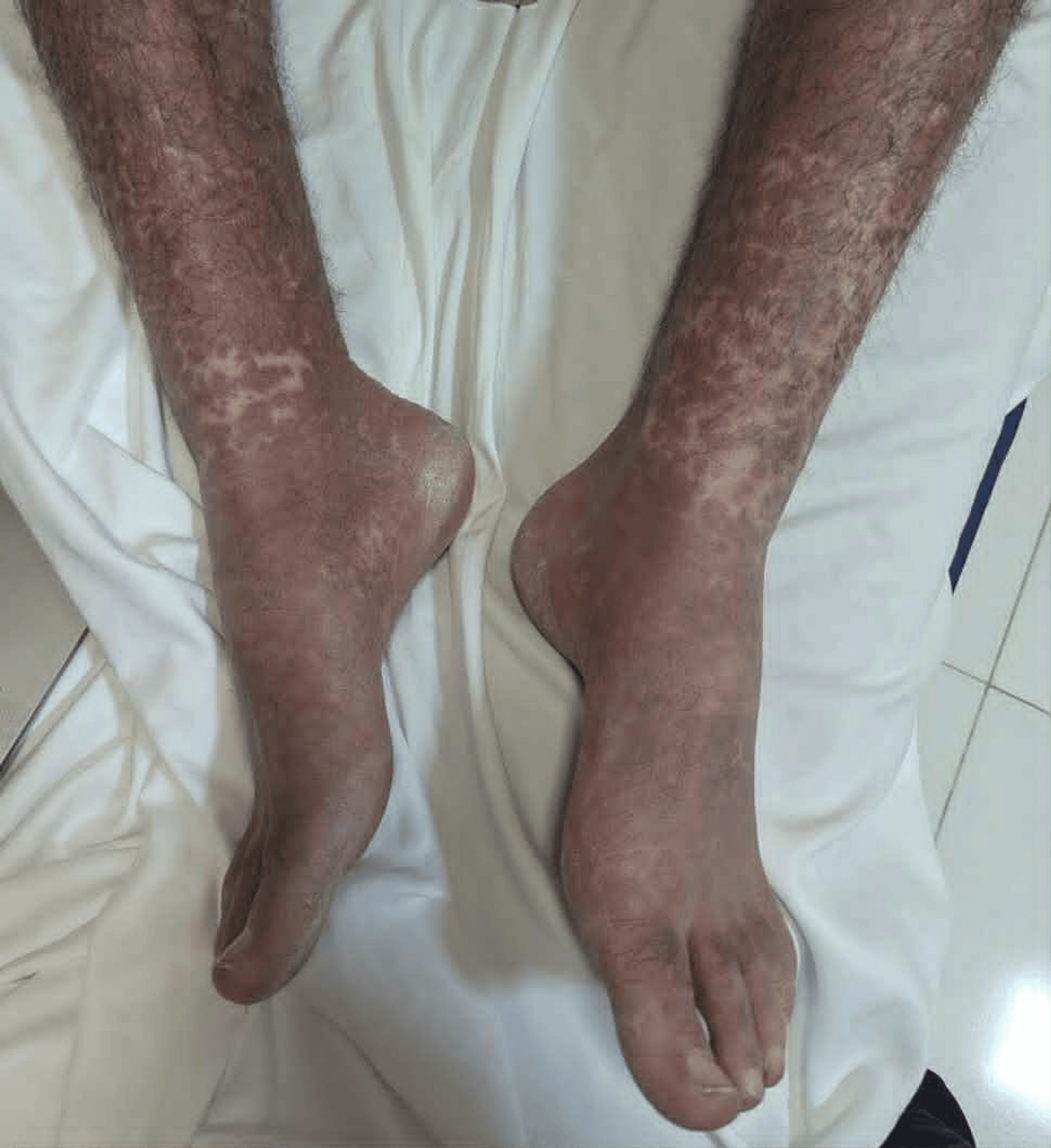
Rash that developed on the lower limps Erythematous maculopapular rash observed on the lower limbs in a 24-year-old male patient diagnosed with infectious mononucleosis caused by the Epstein-Barr virus (EBV)

**Figure 3:**
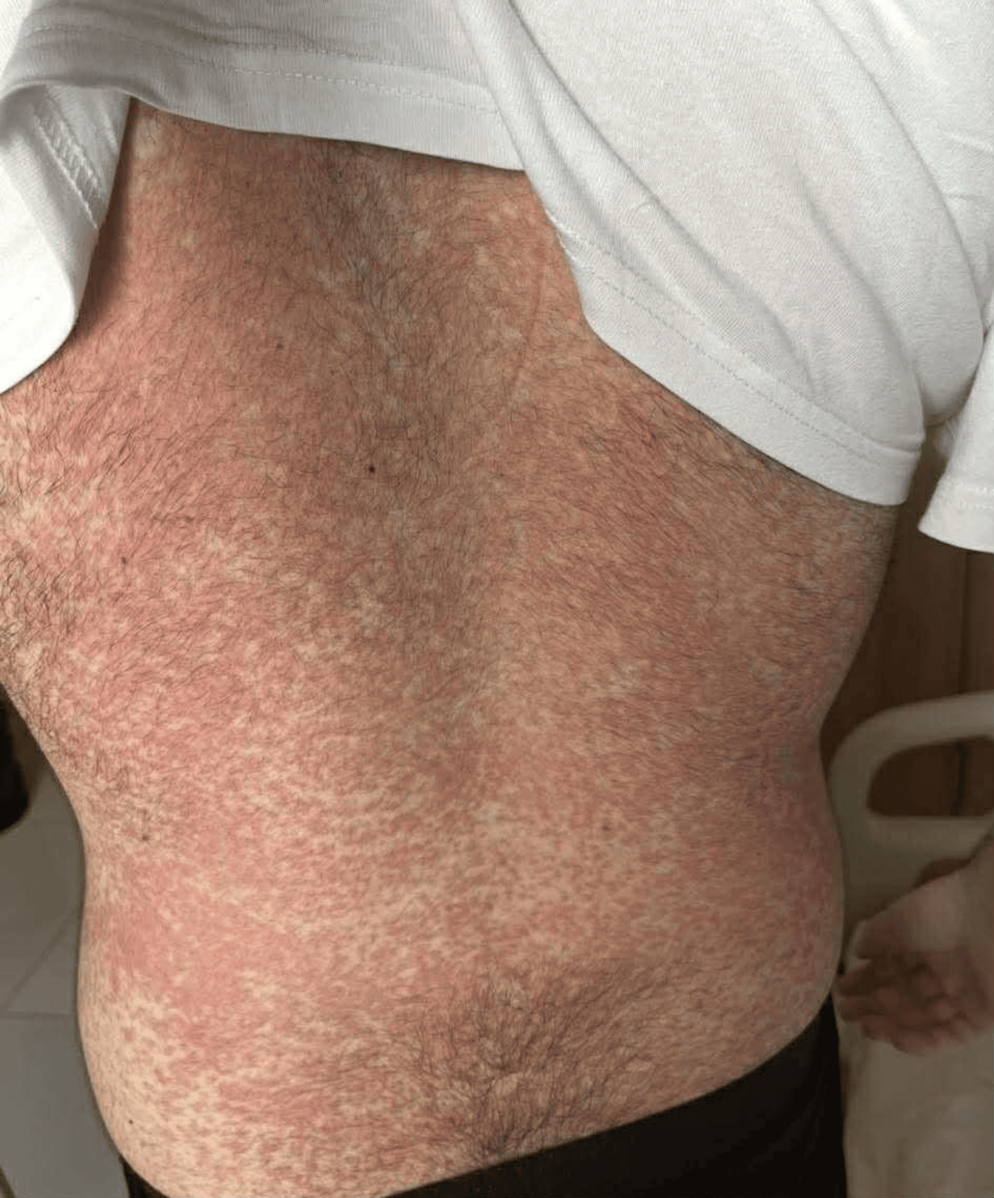
Maculopapular rash developing on the body Maculopapular rash observed on the body in a 24-year-old male patient diagnosed with infectious mononucleosis caused by the Epstein-Barr virus (EBV)

**Figure 4:**
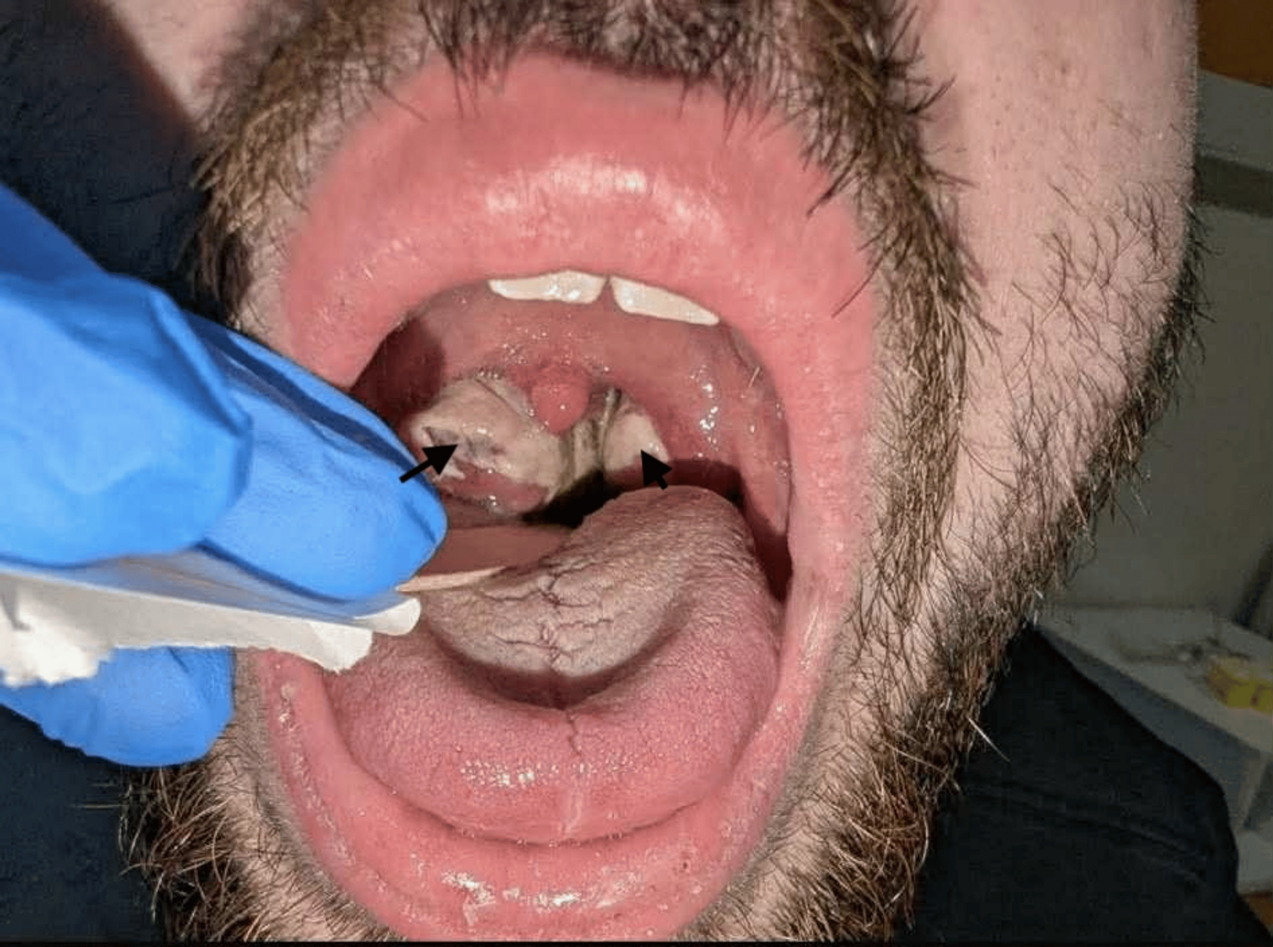
Swollen tonsils covered with exudates Arrows show enlarged, exudate-covered tonsils

Upon hospital admission, physical examination revealed the patient had a high-grade fever (39.5°C), noticeable weakness, and adynamia. Consciousness was clear, adequate, and oriented to time and space; no motor deficits, pathological reflexes, or meningeal signs were identified. Oxygen saturation (SpO_2_) on ambient air was 94%. Heart rate (HR) was 115 beats per minute, pulse was regular, respiratory rate (RR) was 20-21 breaths per minute, and blood pressure (BP) was 115/75 mmHg. Head, eyes, ears, nose, and throat (HEENT) examination showed enlarged tonsils with exudates (Brodsky grade 2), an erythematous pharynx, and tender cervical lymphadenopathy. A diffuse erythematous maculopapular rash was evident on the trunk, face, and upper and lower extremities (see Figure 1-4). 

Respiratory assessment at this stage revealed no signs of distress. Imaging studies revealed several findings. A chest X-ray showed no pathological focal or infiltrative changes. Echocardiography demonstrated preserved left ventricular systolic function (ejection fraction (EF) = 57%) and a small amount of pericardial effusion, approximately 1 cm. Neck lymph node ultrasound identified bilateral enlarged lymph nodes; however, their normal shape was maintained, with sizes ranging from 30 to 36 mm. Abdominal ultrasound, specifically examining the liver and spleen, revealed mild hepatomegaly (right lobe 167 mm, left lobe 98 mm), and the spleen measured 164 x 62 mm. A rapid streptococcal antigen detection test (RADT) was performed, and a throat swab was taken for bacterial culture. The RADT result was negative, and the subsequent culture yielded no growth of Streptococcus pyogenes or other bacterial pathogens.

Concurrently, comprehensive laboratory investigations revealed leukocytosis, lymphocytosis, monocytosis, thrombocytopenia, and markedly elevated lactate dehydrogenase (LDH) and ferritin levels. Liver function tests indicated extremely elevated alanine aminotransferase (ALT), which exceeded quantifiable limits due to its high concentration. Aspartate aminotransferase (AST) was elevated ninefold, accompanied by markedly increased gamma-glutamyl transferase (GGT), alkaline phosphatase (ALP), and total bilirubin, with elevated conjugated bilirubin levels. Furthermore, a significant elevation in ammonia levels was noted. Serological testing ultimately confirmed the diagnosis of EBV infection, demonstrating positive results for EBV-VCA IgM and heterophile antibodies (laboratory data obtained at the time of hospital admission are presented in Table 1). 

**Table 1:** Laboratory data at the time of hospital admission Note: CBC: complete blood count; WBC: white blood cell; RBC: red blood cell; HGB: hemoglobin; PLT: platelet; CRP: C-reactive protein; LDH: lactate dehydrogenase; ALT: alanine aminotransferase; AST: aspartate aminotransferase; GGT: gamma-glutamyl transferase; ALP: alkaline phosphatase; ALB: albumin; NH3: ammonia; HBsAg: hepatitis B surface antigen; anti-HCV: antibodies to hepatitis C virus; anti-HIV 1+2: antibodies to human immunodeficiency virus type 1 and type 2; anti-*Treponema pallidum*: antibodies to *Treponema pallidum* (causative agent of syphilis); EBV-VCA IgM: Epstein-Barr virus - viral capsid antigen IgM; EBV-VCA IgG: Epstein-Barr virus - viral capsid antigen IgG; EBV-EBNA IgG: Epstein-Barr virus - Epstein-Barr nuclear antigen IgG; CMV IgG: cytomegalovirus immunoglobulin G; CMV IgM: cytomegalovirus immunoglobulin M; IU/mL: international units per milliliter

Category	Test	Result	Reference range
Laboratory tests	CBC (hematogram)		
	WBC	18.2 x10^9/L	4.0 - 11.0 x 10^9^/L
	Neutrophils	31.5%	40-70%
	Lymphocytes	55.4%	20-40%
	Monocytes	11.2%	2-8%
	Eosinophils	1.00%	1-4%
	Basophils	0.90%	0-2%
	RBC	4.89 x10^12/L	4.2-5.9 x 10^12^/L
	HGB	14.8	12.0-16.0 g/dL
	PLT	142 x10^9/L	150-450 x10^9/L
Biochemical tests	CRP	13.1	<5.0 mg/L
	LDH	883	125-220 U/L
	Ferritin	1155.6	30-400 ng/mL
	ALT	Elevated beyond measure	7-56 U/L
	AST	358	10-40 U/L
	GGT	219	9-48 U/L
	ALP	489.6	44-147 U/L
	Total bilirubin	36.30	5.0-21.0 µmol/L
	Direct bilirubin	24.96	0-6.8 µmol/L
	Albumin (ALB)	33	35-50 g/L
	Ammonia (NH3)	257	15-45 µg/dL
Serology	HBsAg, anti-HCV, anti-HIV 1+2, anti-treponema pallidum	Negative	Negative
	EBV-VCA IgM	Positive	Negative (varies by lab)
	EBV-VCA IgG	Positive	Positive (varies by lab)
	EBV-EBNA IgG	Negative	Negative
	Heterophile antibodies	Positive	Negative
	CMV IgG	157.5	5.0-50.0 IU/mL
	CMV IgM	0.97 (borderline)	<1.1 IU/mL (borderline)

Given the confirmed acute EBV infection through clinical and laboratory investigations, the temporal correlation between symptom worsening and the initiation of antibiotic therapy, and the negative streptococcal test result, the diagnosis of bacterial tonsillitis was excluded, leading to the discontinuation of antibiotic therapy.

During the initial 24 hours of hospitalization, the patient received intravenous infusion and symptomatic therapy, including acetaminophen and nonsteroidal antiinflammatory drugs (NSAIDs), under the supervision of an otorhinolaryngologist. Despite this ongoing regimen, the patient’s condition did not improve; he continued to experience dysphagia, dyspnea, and a sensation of choking. Upon ear, nose, and throat (ENT) examination, significant bilateral tonsillar enlargement, graded as Brodsky 3, was noted, with associated uvular displacement, indicating a high risk of upper airway obstruction. 

Within the first 24 hours of hospitalization, the patient's condition worsened, manifesting as stridor, cyanosis, and tachypnea. At this point, HR increased to 120 beats per minute, RR was 24 breaths per minute, and SpO_2_ dropped to 91% on ambient air, necessitating oxygen therapy via nasal cannula. The patient was profoundly adynamic, although consciousness remained clear and adequate, with delayed responses to questions; no pathological or meningeal signs were present. Given the escalating risk of developing severe upper airway obstruction, it was deemed imperative to incorporate systemic glucocorticosteroids (dexamethasone 6 mg) into the treatment regimen. While upper airway compromise due to tonsillar hypertrophy is uncommon in IM, systemic corticosteroids are a recognized treatment for this complication during hospitalization [9]. Crucially, despite the severity of airway compromise, the patient did not require intubation.

Within 48 hours of initiating this revised treatment, the patient’s clinical condition began to improve, with febrile episodes becoming less frequent and the rash starting to regress. By the sixth day of hospitalization, laboratory findings showed significant improvement (Table 2). 

**Table 2:** Following treatment with dexamethasone Note: CBC: complete blood count; WBC: white blood cell; PLT: platelet; LDH: lactate dehydrogenase; NH3: ammonia; ALT: alanine aminotransferase; AST: aspartate aminotransferase; GGT: gamma-glutamyl transferase; ALP: alkaline phosphatase

Category	Test	Result	Reference range
Laboratory tests	CBC (hematogram)		
	WBC	14.5 x 10^9^/L	4.0-11.0 x 10^9^/L
	Neutrophils	61.9%	40-70%
	Lymphocytes	30.50%	20-40%
	PLT	190 x 10^9^/L	150-450 x10^9/L
Biochemical tests	LDH	415.5	125-220 U/L
	Ammonia (NH3)	40	15-45 µg/dL
	ALT	65	7-56 U/L
	AST	33	10-40 U/L
	GGT	55	9-48 U/L
	ALP	60	44-147 U/L
	Total bilirubin	15	5.0-21.0 µmol/L
	Direct bilirubin	5	0-6.8 µmol/L
	Ferritin	510	30-400 ng/mL

**Figure 5:**
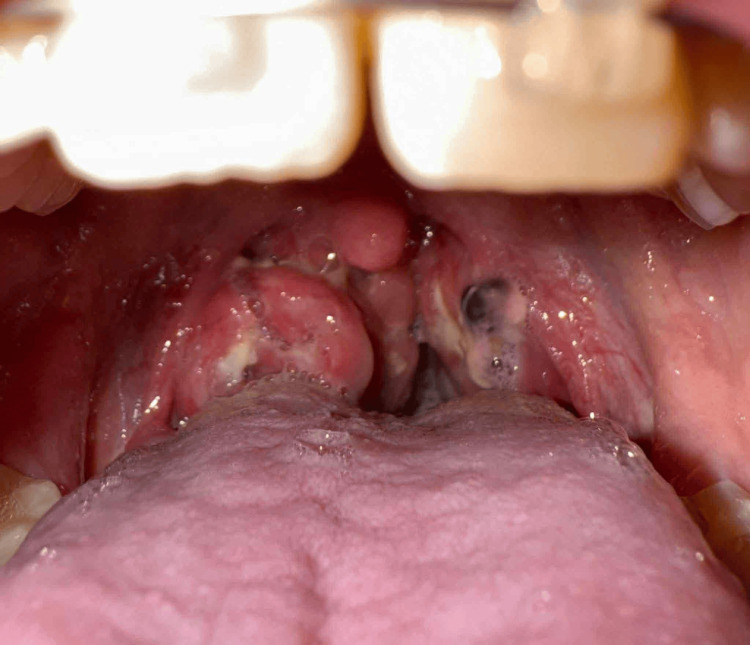
Tonsils after treatment The tonsillar exudate resolved and tonsillar swelling decreased.

The rash had markedly diminished, and both tonsillar exudates and swelling had resolved (see Figure 5). 

Upon discharge, the patient was afebrile, vital signs were within normal limits (e.g., HR 87 bpm, RR 18 bpm, BP 110/70 mmHg, SpO_2_ 98%), and tonsillar hypertrophy had significantly regressed. Laboratory parameters, including liver function tests and complete blood count, showed continued improvement, approaching baseline levels. The patient reported resolution of dysphagia and dyspnea, with only mild residual fatigue. The patient was subsequently discharged in improved clinical condition.

## Discussion

This case report meticulously details the presentation and management of a 24-year-old male with severe IM whose initial clinical picture was complicated by a misdiagnosis of bacterial tonsillitis and subsequent amoxicillin-clavulanic acid administration. The constellation of symptoms, including high fever, odynophagia, and cervical lymphadenopathy, as initially observed in our patient, is frequently shared between IM and bacterial pharyngitis, presenting a well-recognized diagnostic dilemma in clinical practice [5]. This diagnostic challenge often culminates in the unnecessary prescription of antibiotics, particularly aminopenicillins, to patients with IM, as was the situation in our case [8].

A cardinal feature complicating our patient's course was the development of a diffuse maculopapular rash shortly after initiating amoxicillin. While this finding, characterized by its non-IgE-mediated mechanism, is a well-documented hypersensitivity reaction in EBV-infected individuals rather than a true drug allergy [10], its appearance significantly obscured the underlying viral etiology and underscored the initial diagnostic uncertainty. The high reported incidence of such rashes, ranging from 27.8% to 69% in EBV patients treated with aminopenicillins [11], further emphasizes the importance of considering IM in the differential diagnosis of pharyngitis, especially prior to antibiotic prescription.

The initial diagnostic process focused on differentiating the viral etiology from bacterial infections, particularly streptococcal pharyngitis. Despite the classic pharyngeal and systemic symptoms, rapid streptococcal antigen detection tests and subsequent throat culture consistently yielded negative results, effectively ruling out GABHS as the primary pathogen [12]. Furthermore, other potential causes of similar widespread rashes and systemic symptoms, such as measles and scarlet fever, were considered and excluded. The patient's rash, while maculopapular, did not exhibit the characteristic features of measles (e.g., cephalocaudal spread) [13] or scarlet fever (e.g., "sandpaper" texture, Pastia's lines, "strawberry tongue") [14]. The progression of our patient's symptoms to severe odynophagia, dysphagia, dyspnea, and a sensation of choking necessitated hospitalization and intensive monitoring. Upper airway obstruction is a well-documented complication of IM [15]. Upon ENT examination, significant bilateral tonsillar enlargement, graded as Brodsky 3, was observed, with kissing tonsils that nearly met in the midline, causing significant narrowing of the oropharynx. This critical finding prompted immediate intervention with intravenous corticosteroids, a mainstay in reducing lymphoid tissue hypertrophy and alleviating airway obstruction in IM [16].

Following the initiation of intravenous dexamethasone at a daily dose of 6 mg, the patient demonstrated a remarkable clinical improvement within 48 hours. His odynophagia and dysphagia significantly subsided, respiratory distress resolved, and tonsillar swelling markedly reduced, allowing for improved oral intake and comfort. Supportive care, including intravenous infusion, acetaminophen, and NSAIDs, was continued during this period. The diagnosis of IM was further confirmed by positive EBV IgM viral capsid antigen (VCA) antibodies and positive heterophile antibodies, reinforcing the viral etiology and the appropriateness of the corticosteroid intervention for the airway compromise.

## Conclusions

IM continues to present a diagnostic challenge in clinical practice, often mimicking bacterial pharyngitis and leading to the unnecessary administration of antibiotics. This case highlights the importance of maintaining a high index of suspicion for IM, particularly in patients with negative streptococcal tests and atypical presentations. The development of an aminopenicillin-induced rash serves as a crucial indicator for re-evaluating the initial diagnosis and considering IM. Furthermore, this report underscores airway compromise as a rare but severe complication of IM, emphasizing the need for prompt recognition and effective management with corticosteroids. Ultimately, this case advocates for a vigilant and nuanced approach to pharyngitis, promoting judicious antibiotic use and ensuring timely intervention for potentially life-threatening complications of IM.
